# National survey on deceased donor organ transplantation during the COVID-19 pandemic in Japan

**DOI:** 10.1007/s00595-021-02388-1

**Published:** 2021-10-23

**Authors:** Taihei Ito, Takashi Kenmochi, Atsuhiko Ota, Kaori Kuramitsu, Akihiko Soyama, Osamu Kinoshita, Susumu Eguchi, Kenji Yuzawa, Hiroto Egawa

**Affiliations:** 1 Department of Transplantation and Regenerative Medicine, Fujita Health University, School of Medicine, Dengakugakubo 1-98, Kutsukakecho, Toyoake, Aichi 470-1192 Japan; 2 Department of Public Health, Fujita Health University School of Medicine, Toyoake, Aichi Japan; 3 Division of Hepato-Biliary-Pancreatic Surgery, Kobe University Graduate School of Medicine, Kobe, Hyogo Japan; 4 Department of Surgery, Nagasaki University Graduate School of Biomedical Sciences, Nagasaki, Nagasaki Japan; 5 Department of Cardiac Surgery, Graduate School of Medicine, The University of Tokyo, Bunkyo, Tokyo Japan; 6 Department of Transplantation Surgery, National Hospital Organization Mito Medical Center, Mito, Ibaraki Japan; 7 Department of Surgery, Institute of Gastroenterology, Tokyo Women’s Medical University, Shinjuku, Tokyo Japan

**Keywords:** COVID-19, Brain-dead donors, Donors after cardiac death, Solid organ transplantation

## Abstract

**Purpose:**

We investigated the status of deceased organ donation and transplantation through a questionnaire distributed to transplant centers in Japan during the COVID-19 pandemic.

**Methods:**

The questionnaire was distributed electronically to 206 transplant centers for heart (*n* = 11), lung (*n* = 10), liver (*n* = 25), kidney (*n* = 130), pancreas (*n* = 18), and small intestine (*n* = 12) transplantation. Organ donations and organ transplantation data were extracted from the Japan Organ Transplant Network website.

**Results:**

We received questionnaire responses from 177 centers (response rate, 86%). In 2020, the number of brain-dead donors (BDDs) decreased to 68 (69% of the year-on-year average) and the number of donors after cardiac death (DCDs) decreased to 9 (32% of the year-on-year average). Eighty-five (48%) transplant centers (heart, *n* = 0; lung, *n* = 0; liver, *n* = 4; kidney, *n* = 78; pancreas, *n* = 22; and small intestine, *n* = 0) suspended transplant surgeries in response to the COVID-19 pandemic. Consequently, the number of organ transplantations from deceased donors was significantly lower in 2020 than in 2019.

**Conclusion:**

Although the COVID-19 pandemic has had less impact in Japan than in other countries, it has affected transplantation activity significantly, suspending transplantation surgeries in 48% of the transplantation centers, including 78% of the kidney transplantation centers, and reducing the number of organ donations to 61% of the year-on-year average.

## Introduction

In the first 10 years after the establishment of the Brain Death Organ Transplant Act in 1997, the annual number of brain-dead donors (BDDs) in Japan was less than 10. This low number at the time was attributed to the fact that brain death referred only to human death in cases of donation for organ transplantation, and brain-dead donors (BDDs) were required to have made their intention clear before brain death. The law was revised in 2009 to state that even if the donor's intention to donate organs was unknown, organ donation from BDDs was able to be approved with the consent of the family. The law was enforced in 2010, resulting in increased numbers of organ donations from BDDs [1]. This had a positive impact on patients awaiting solid organ transplantation (SOT) for organ failure [2–6].

The COVID-19 pandemic has ravaged the globe since early 2020 [7–11]. While the numbers of patients with COVID-19 and related deaths in Japan have been relatively low in comparison with other countries [12, 13], the treatment of COVID-19 patients in emergency medical care and the intensive-care unit (ICU) has been difficult, raising concern about the decline of general medical practices [14–16].

In transplant medical care, essential immunosuppressive therapy after transplantation has a negative impact on mortality from this new viral infection. The COVID-19-related mortality rate in SOT recipients has been reported to range from 18 to 34% [17–19]. Moreover, COVID-19 is a serious life-threatening infectious disease for individuals with organ failure who are awaiting transplantation, and the decline in transplant activity also impacts the prognosis of these patients. For example, the COVID-19-related mortality rate in patients awaiting kidney transplantation who required dialysis was reported to range from 20 to 32% [18, 20–32].

In response to the impact of the COVID-19 pandemic on transplant activity, on March 6, 2020, the Japan Society for Transplantation published the first edition of the basic guidelines for transplantation medicine for new coronavirus infection (COVID-19). The guidelines have been updated according to the outbreak situation in Japan, with the latest edition, version 4.1 released on February 4, 2021 [33]. In the first edition, the recommendation for the implementation of SOT was as follows: “If possible, waiting for organ transplantation until the situation of the COVID-19 pandemic improves is recommended in order to avoid the risk of infection from donors or community-acquired infection under immunosuppression after transplantation”. However, the recommendation in the latest version (version 4.1) is as follows: “In the implementation of solid organ transplantation, the risk of the COVID-19 infection from the donor and the risk of the aggravation of COVID-19 infection under immunosuppression after transplantation must be properly explained to obtain sufficient informed consent about…”. Thus, although the COVID-19 pandemic may influence organ donation and transplant activity in Japan remarkably, no report has clarified the real situation.

We investigated the status of deceased organ donation and organ transplantation in Japan during the COVID-19 pandemic, using a questionnaire that was distributed to transplant centers, to clarify the current status of transplantation activity in Japan during the COVID-19 pandemic.

## Methods

A questionnaire survey was conducted as part of a welfare and labor science special research project entitled, “Survey research for organ transplantation from BDDs and donors after cardiac death (DCDs) during the pandemic of COVID-19”. The questionnaire, including 23 questions on the medical system, the SOT policy, and the implementation status in each center during the COVID-19 pandemic, was distributed electronically to 206 transplant centers in Japan (heart centers, *n* = 11; lung centers, *n* = 10; liver centers, *n* = 25; kidney centers, *n* = 130; pancreas centers, *n* = 18; and small-intestine centers, *n* = 12) from December, 2020 to January, 2021 during the third wave of the COVID-19 pandemic, with the development of a website entrusted to Tokai Kyodo Printing. The representatives of each center registered their answers. The numbers of deceased organ donations and SOTs were counted by searching the Japan Organ Transplant Network website (https://www.jotnw.or.jp/). The number of COVID-19-positive patients in Japan was estimated based on announcements made by the Ministry of Health, Labor and Welfare　(https://www.mhlw.go.jp/stf/covid-19/kokunainohasseijoukyou.html).

The Microsoft Excel software program (Microsoft, USA) was used to summarize the data. Categorical data are shown as frequencies and percentages.

## Results

### Changes in the number of COVID-19-positive patients and organ donations in Japan

**Fig. 1:**
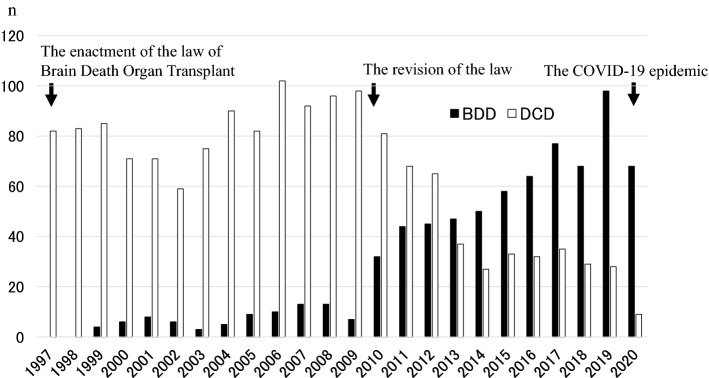
Annual numbers of deceased organ donations in Japan. Organ donations from brain-dead donors (BDDs) (black solid bars) began in 1997, when the law on organ transplantation was enacted. When the law was revised in 2009, the number of brain-dead organ donations increased steadily, reaching a record high of 98 cases in 2019. In contrast, the number of donors after cardiac death (DCDs) (white solid bars) has decreased since the revision of the law, falling to approximately 30 in recent years. In 2020, after detection of the first COVID-19-positive patients in Japan, the number of BDDs and DCDs decreased to 68 and 9, respectively

Figure 1 shows the changes in the number of BDDs and DCDs in Japan. In 1997, the law on organ transplantation was enacted, with organ donations from BDDs (black solid bars) implemented. Despite this legislation, there was no marked increase in organ donations from BDDs. The law was revised in 2009 and enforced in 2010, after which the number of brain-dead organ donations began to steadily increase, reaching a record high of 98 in 2019. In contrast, the number of DCDs (white solid bars) has decreased since the revision of the law, to about 30 in recent years. In early 2020, the first cases of COVID-19 were detected in Japan, and in 2020, the number of BDDs and DCDs decreased to 68 and 9, respectively.

**Fig. 2:**
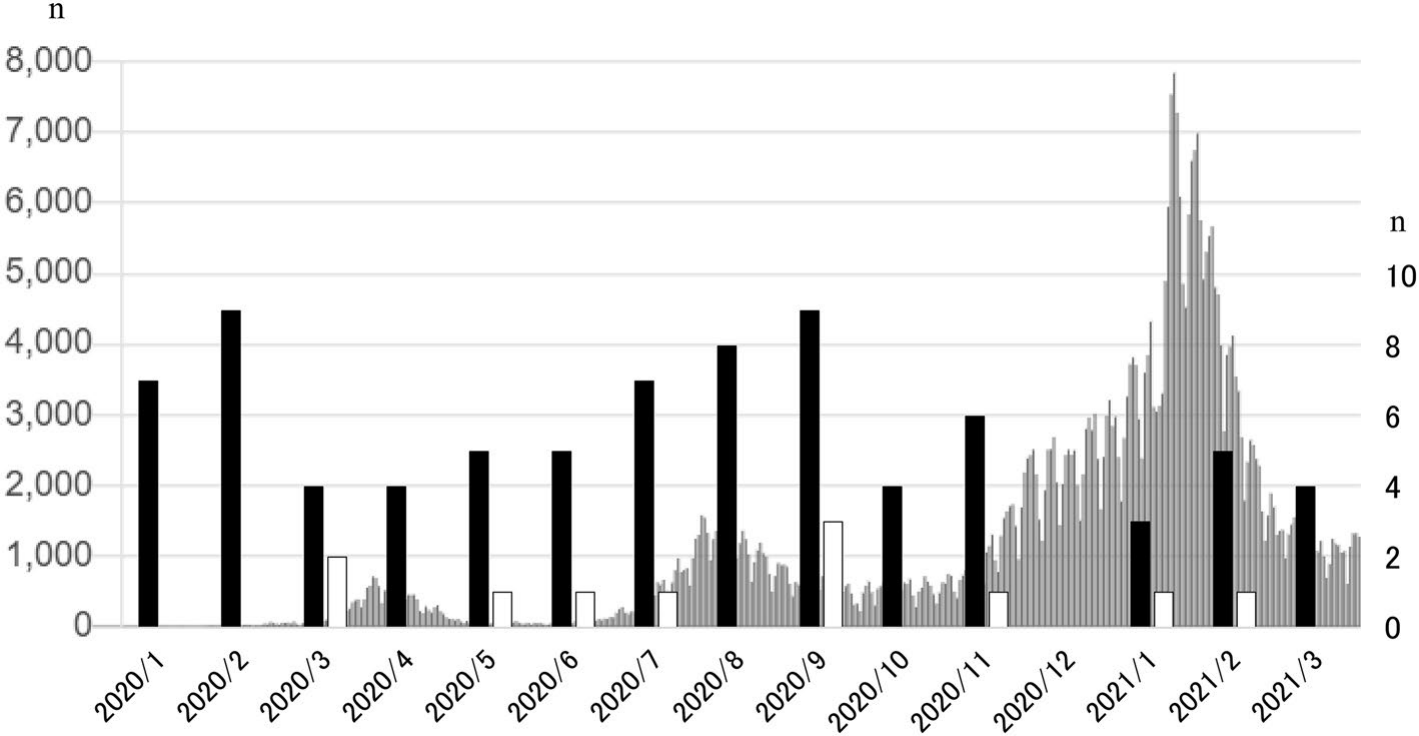
Numbers of COVID-19-positive patients and deceased donors in 2020. The change in the number of COVID-19-positive patients is displayed as solid gray bars, and the monthly numbers of organ donations from BDDs and DCDs since January 2020 are shown as solid black bars and solid white bars, respectively. Since the autumn of 2020, when the third wave of the COVID-19 pandemic started, the number of organ donations has decreased. In December 2020, there were no organ donations. It is noteworthy that the number of DCDs, which require a long waiting period for transplant doctors, has dropped significantly since early 2020

Figure 2 shows the changes in the number of COVID-19-positive patients (gray solid bar) and the monthly number of organ donations (BDDs, black solid bars; DCDs, white solid bars) from January, 2020. The first case of COVID-19 in Japan was confirmed in February, 2020, with the number peaking in April, 2020 in the first wave of the COVID-19 pandemic. This was followed by a second wave in August, 2020, and a third wave in February, 2021. By the time of writing, at the end of March 2021, the third wave had begun to converge. By March 20, 2021, the total number of COVID-19 positive patients in Japan had reached 451,830, with 8788 COVID-19-related deaths.

In contrast, the number of BDDs at the beginning of 2020 did not decrease significantly from that in 2019. However, since the autumn of 2020, when the third wave of the COVID-19 pandemic started, the number of organ donations has decreased, with no organ donations in December, 2020. It is noteworthy that the number of DCDs, which require a long waiting period for transplant doctors, has dropped significantly since early 2020, to only nine cases in 2020. This represents the first single-digit figure in the records, and highlights the significant impact of the COVID-19 pandemic on organ donation in Japan (Fig. 2).

**Fig. 3:**
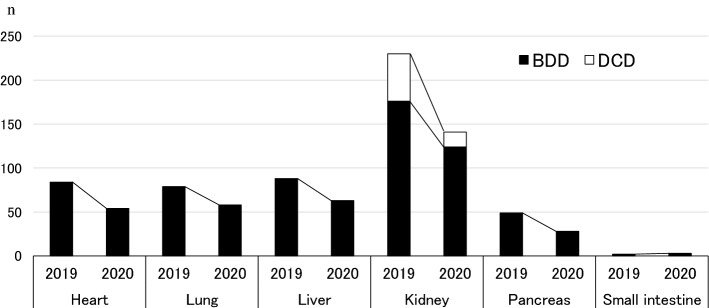
Annual numbers of transplantations from deceased donors in 2019 and 2020 in Japan. There were 84 cases of heart transplantation in 2019 vs. 54 in 2020 (64% of the year-on-year average) and 79 cases of lung transplantation in 2019 vs. 58 in 2020 (73% of the year-on-year average). For abdominal organ transplantation, there were 88 cases of liver transplantation in 2019 vs. 63 in 2020 (72% of the year-on-year average) and 176 cases of kidney transplantation, including 46 of simultaneous pancreas transplantation (SPK) and 6 of simultaneous liver transplantation (SLK), in 2019 vs. 124 (SPK, *n* = 24; SLK, *n* = 5; 71% of the year-on-year average) in 2020. Pancreas transplants also decreased from 49 cases in 2019 to 28 in 2020 (57% of the year-on-year average). Small-intestine transplants increased slightly from two cases in 2019 to three in 2020

As a result, the annual number of SOTs from deceased donors decreased significantly in 2020 compared with the number in 2019, with the exception of small-intestine transplants (Fig. 3). There were 84 cases of heart transplantation in 2019 vs. 54 in 2020 (64% of the year-on-year average) and 79 cases of lung transplantation in 2019 vs. 58 in 2020 (73% of the year-on-year average). Regarding abdominal organ transplantation, there were 88 cases of liver transplantation in 2019 vs. 63 in 2020 (72% of the year-on-year average) and 176 cases of kidney transplantation, including 46 cases of simultaneous pancreas transplantation (SPK) and 6 cases of simultaneous liver transplantation (SLK), in 2019 vs. 124 in 2020 (SPK, *n* = 24; SLK, *n* = 5; 71% of the year-on-year average). Pancreas transplants also decreased from 49 cases in 2019 to 28 in 2020 (57% of the year-on-year average), whereas small-intestine transplants increased slightly from 2 cases in 2019 to 3 in 2020.

### Current status of transplant medical care at transplant centers in Japan

We received questionnaire responses from 177 of the 206 centers, representing a response rate of 86% (heart centers, 100%; lung centers, 90%; liver centers, 100%; kidney centers, 79%; pancreas centers, 100%; small-intestine centers, 92%).

Table 1 summarizes the medical care for COVID-19 (other than transplants) that was provided in transplant centers. Among the responding centers, 98 (55%) were designated hospitals for infection (Q1), and 155 (88%), including those other than designated hospitals for infection, had wards dedicated to the treatment of COVID-19-positive patients (Q3). ICUs were set up in-hospital at 172 centers (97%) (Q4), and patients with severe COVID-19 sequelae were treated in the ICU at 144 centers (81%) (Q5). Regarding in-hospital tests for COVID-19, PCR testing was performed at 169 centers (95%) (Q6), antigen tests were performed at 151 centers (85%) (Q7), and antibody tests were performed at 90 centers (51%) (Q8). A total of 161 centers (90%) had established rules on surgical treatment (including surgery other than transplantation) during the COVID-19 pandemic (Q9).

**Table 1:** Medical care for COVID-19 in transplant centers

		Answer	Overall (%) *n* = 177	Heart *n* = 11	Lung *n* = 9	Liver *n* = 25	Kidney *n* = 103	Pancreas *n* = 18	Small intestine *n* = 11
Q1	Is your center an infectious disease designated hospital?	Yes	98 (55)	2 (18)	6 (67)	18 (72)	52 (50)	12 (67)	8 (73)
		No	79 (45)	9 (82)	3 (33)	7 (28)	51 (50)	6 (33)	3 (27)
Q2	Do you have a special outpatient clinic that provides care for patients with fever in your hospital?	Yes	127 (72)	5 (45)	6 (67)	18 (72)	79 (77)	12 (67)	7 (64)
		No	49 (28)	6 (55)	3 (33)	7 (28)	23 (22)	6 (33)	4 (36)
Q3	Do you have a ward dedicated to treating COVID-19 infected patients in your hospital?	Yes	155 (88)	11 (100)	9 (100)	20 (80)	90 (87)	17 (94)	8 (73)
		No	22 (12)	0 (0)	0 (0)	5 (20)	13 (13)	1 (6)	3 (27)
Q4	Do you have an ICU in your hospital?	Yes	172 (97)	11 (100)	9 (100)	25 (100)	98 (95)	18 (100)	11 (100)
		No	5 (3)	0 (0)	0 (0)	0 (0)	5 (5)	0 (0)	0 (0)
Q5	Does the ICU accept treatment for patients infected with COVID-19?	Yes	144 (81)	11 (100)	9 (100)	23 (92)	76 (74)	15 (83)	10 (91)
		No	32 (19)	0 (0)	0 (0)	2 (8)	26 (25)	3 (17)	1 (9)
Q6	Is it possible to perform PCR tests in-hospital for COVID-19 in your center?	Yes	169 (95)	11 (100)	9 (100)	25 (100)	95 (92)	18 (100)	11 (100)
		No	7 (4)	0 (0)	0 (0)	0 (0)	7 (7)	0 (0)	0 (0)
Q7	Is it possible to perform antigen tests in-hospital for COVID-19 in your center?	Yes	151 (85)	9 (82)	8 (89)	20 (80)	90 (87)	16 (89)	8 (73)
		No	21 (12)	0 (0)	1 (11)	3 (12)	13 (13)	2 (11)	2 (18)
Q8	Is it possible to perform antibody tests in-hospital for COVID-19 in your center?	Yes	90 (51)	8 (73)	6 (67)	14 (56)	46 (45)	10 (56)	6 (55)
		No	78 (44)	2 (18)	3 (33)	9 (36)	54 (52)	7 (39)	3 (27)
Q9	Do you have any rules related to COVID-19 concerning the performance of surgical treatment (including surgery other than transplantation) in your center?	Yes	161 (90)	9 (82)	9 (100)	23 (92)	94 (91)	17 (94)	9 (82)
		No	12 (7)	0 (0)	0 (0)	1 (4)	8 (8)	1 (6)	2 (18)

**Fig. 4:**
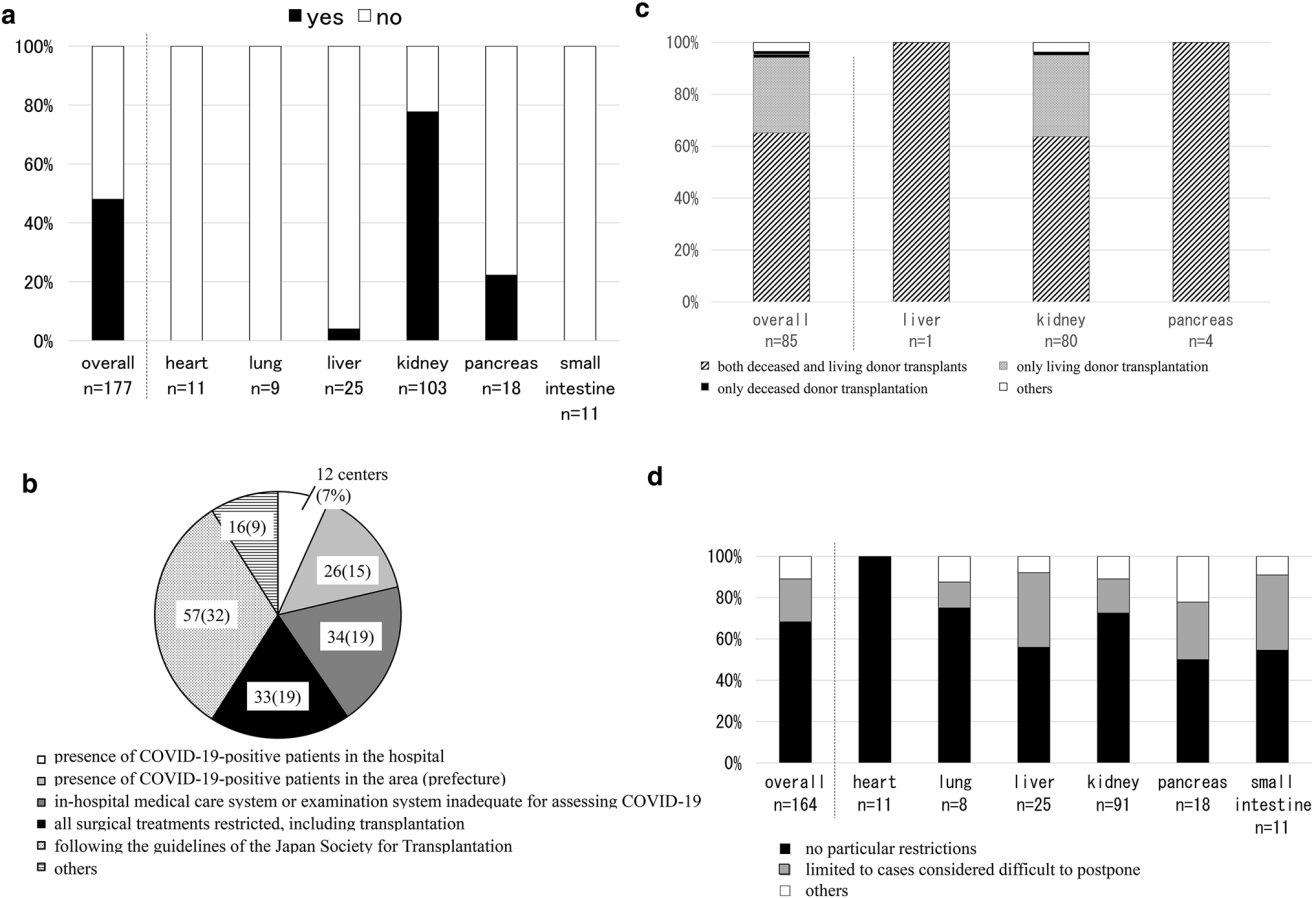
Answers to questions about porting program continuation. Q12: Have organ transplant surgeries in your hospital been stopped due to the COVID-19 pandemic? (**a**), Q13: What was the reason for the decision to suspend organ transplant surgeries? (Multiple answers allowed) (**b**), Q14: What kind of organ transplants were suspended? (**c**), Q16: What kind of organ transplants are being performed if transplant surgery is ongoing? (**d**). Response summaries: Q12: While no center discontinued its transplant surgeries for heart and lung transplants, one center (4%) suspended its transplant surgeries for liver transplants, 80 (78%) suspended their transplant surgeries for kidney transplants, and 4 (22%) suspended their transplant surgeries for pancreas transplants (**a**). Q13: The reasons for discontinuing transplantation were as follows: COVID-19-positive patients in the hospital (*n* = 12; 7%), COVID-19-positive patients in the prefecture (*n* = 26; 15%), in-hospital medical care system or examination system inadequate for assessing COVID-19 (*n* = 34; 19%), all surgical treatment (including transplantation) restricted (*n* = 33; 19%), and suspended in accordance with guidelines of the Japan Society for Transplantation (*n* = 57; 32%) (**b**). Q14: Fifty-six centers (31%) discontinued all transplants, including both deceased and living donor transplantation, 25 (14%) discontinued only living donor transplantation, and 1 (1%) discontinued only deceased donor transplantation (**c**). Q16: Of the centers with an ongoing transplant surgeries at the time of the questionnaire, 112 (63%) were providing transplant medical care without any specific restrictions, while 34 (19%) were limiting transplant surgery to cases when it was considered difficult to postpone (**d**)

Table 2 summarizes the results of the questionnaire on the status of transplant programs at transplant centers in Japan during the COVID-19 pandemic. Regarding the continuation of providing transplant medical care, following discussions held at 151 centers (85%) (Q10), 85 centers (48%) suspended transplant medical care because of the COVID-19 pandemic (Q12). While no center discontinued its transplant surgery for heart and lung transplants, one center (4%) suspended liver transplants, 80 (78%) suspended kidney transplants, and 4 (22%) suspended pancreas transplants (Fig. 4a). In 41 centers (23%), this discussion involved a single department, in 57 (32%) it involved multiple departments concerned with SOT, and in 44 (25%) it involved the whole hospital (Q11). The reasons for suspending transplant surgeries (Q13) were as follows: there were COVID-19-positive patients in the hospital (*n* = 12; 7%), there were COVID-19-positive patients in the prefecture (*n* = 26; 15%), the in-hospital medical care system or examination system was inadequate for assessing COVID-19 (*n* = 34; 19%), all surgical treatment (including transplantation) was restricted (*n* = 33; 19%), and transplant surgery was suspended in accordance with the guidelines of the Japan Society for Transplantation (*n* = 57; 32%) (Fig. 4b). Fifty-six centers (31%) suspended all transplants, including both deceased and living donor transplantation, 25 (14%) suspended only living donor transplantation, and 1 (1%) suspended only deceased donor transplantation (Fig. 4c) (Q14).

**Table 2:** Results of the questionnaire on the status of transplant programs

		Answer	Overall (%) *n* = 177	Heart *n* = 11	Lung *n* = 9	Liver *n* = 25	Kidney *n* = 103	Pancreas *n* = 18	Small intestine *n* = 11
Q10	Have you ever discussed the continuation of the transplant surgeries in the hospital during the COVID-19 pandemic?	Yes	151 (85)	8 (73)	6 (67)	20 (80)	94 (91)	15 (83)	8 (73)
		No	26 (15)	3 (27)	3 (33)	5 (20)	9 (9)	3 (17)	3 (27)
Q11	To what extent did that discussion take place?	Single department	41 (23)	0 (0)	1 (17)	5 (25)	30 (32)	3 (20)	2 (25)
		Multiple department	57 (32)	5 (63)	0 (0)	9 (45)	31 (33)	8 (53)	4 (50)
		All over hospital	44 (25)	3 (38)	5 (83)	5 (25)	25 (27)	4 (27)	2 (25)
		Others	7 (4)	0 (0)	0 (0)	1 (5)	6 (6)	0 (0)	0 (0)
Q12	Have the organ transplant surgeries in your hospital been stopped due to the COVID-19 pandemic?	Yes	85 (48)	0 (0)	0 (0)	1 (4)	80 (78)	4 (22)	0 (0)
		No	92 (52)	11 (100)	9 (100)	24 (96)	23 (22)	14 (78)	11 (100)
Q13	What was the reason for the decision to suspend the organ transplant surgeries? (Multiple answers allowed)	Presence of COVID-19-positive patients after transplantation in the hospital	0 (0)	N/A	N/A	0 (0)	0 (0)	0 (0)	N/A
		Presence of COVID-19-positive patients in the hospital	12 (7)	N/A	N/A	1 (100)	11 (14)	0 (0)	N/A
		Presence of COVID-19-positive patients in the area (prefecture)	26 (15)	N/A	N/A	0 (0)	26 (33)	0 (0)	N/A
		In-hospital medical care system or examination system inadequate for assessing COVID-19	34 (19)	N/A	N/A	1 (100)	33 (41)	0 (0)	N/A
		All surgical treatments restricted, including transplantation	33 (19)	N/A	N/A	1 (100)	30 (38)	2 (50)	N/A
		Following the guidelines of the Japan Society for Transplantation	57 (32)	N/A	N/A	0 (0)	54 (68)	3 (75)	N/A
		Others	16 (9)	N/A	N/A	0 (0)	15 (19)	1 (25)	N/A
Q14	What kind of organ transplants were suspended?	Both deceased donor transplants and living donor transplantation	56 (31)	N/A	N/A	1 (100)	51 (64)	4 (100)	N/A
		Only living donor transplantation	25 (14)	N/A	N/A	0 (0)	25 (31)	0 (0)	N/A
		Only high-risk transplantation including ABO incompatible cases	0 (0)	N/A	N/A	0 (0)	0 (0)	0 (0)	N/A
		Deceased donor transplantation	1 (1)	N/A	N/A	0 (0)	1 (1)	0 (0)	N/A
		Others	3 (2)	N/A	N/A	0 (0)	3 (4)	0 (0)	N/A
Q15	What was the reason for resuming the organ transplant surgeries? (Multiple answers allowed)	Reduction in numbers of COVID-19 infections in the hospital	9 (5)	N/A	N/A	0 (0)	8 (11)	1 (25)	N/A
		A lack of spread of COVID-19 infection confirmed in the hospital	11 (6)	N/A	N/A	1 (100)	8 (11)	2 (50)	N/A
		Reduction in numbers of COVID-19 infections in the local area	29 (16)	N/A	N/A	0 (0)	27 (36)	2 (50)	N/A
		Establishment of an in-hospital medical care system and examination system for COVID-19	51 (29)	N/A	N/A	1 (100)	48 (64)	2 (50)	N/A
		Restrictions on surgeries other than transplantation lifted	28 (16)	N/A	N/A	1 (100)	25 (33)	2 (50)	N/A
		Following the guidelines of the Japan Society for Transplantation	34 (19)	N/A	N/A	0 (0)	32 (43)	2 (50)	N/A
		Others	8 (4)	N/A	N/A	0 (0)	8 (11)	0 (0)	N/A
Q16	What kind of organ transplants are being performed if the program is ongoing?	Without any particular restrictions	112 (63)	11 (100)	6 (67)	14 (56)	66 (69)	9 (50)	6 (55)
		Limited to cases considering being difficult to postpone	34 (19)	0 (0)	1 (11)	9 (36)	15 (16)	5 (28)	4 (36)
		Others	18 (10)	0 (0)	1 (11)	2 (8)	10 (10)	4 (22)	1 (9)
Q17	If the COVID-19 epidemic expands more in the near future, how do you think that organ transplants be carried out in your hospital?	Without any particular restrictions	30 (17)	5 (45)	1 (11)	4 (16)	13 (14)	4 (22)	3 (27)
		Considering discontinuing their transplant surgeries depending on the presence of COVID-19 patients in the ICU	60 (34)	5 (45)	7 (78)	15 (60)	20 (21)	6 (33)	7 (64)
		Considering discontinuing their transplant surgeries depending on the presence of COVID-19 patients in the hospital	32 (18)	1 (9)	0 (0)	6 (24)	19 (20)	3 (17)	3 (27)
		Discontinue the transplant surgeries should nosocomial COVID-19 infection be observed	34 (19)	1 (9)	0 (0)	2 (8)	30 (31)	0 (0)	1 (9)
		Not decided, yet	24 (13)	2 (18)	1 (11)	2 (8)	15 (16)	4 (22)	0 (0)
		Others	23 (13)	0 (0)	0 (0)	3 (12)	16 (17)	4 (22)	0 (0)
Q18	Have you placed any restrictions on the dispatch of organ recovery from your center during the COVID-19 pandemic?	Yes	21 (12)	0 (0)	0 (0)	1 (4)	18 (17)	1 (6)	1 (9)
		No	148	11 (100)	9 (100)	22 (88)	79 (77)	17 (94)	10 (91)
Q19	Have you experienced any cases in which organ recovery was abandoned due to a COVID-19 infection?	Yes	14 (8)	3 (27)	0 (0)	0 (0)	8 (8)	3 (17)	0 (0)
		No	156 (88)	8 (73)	9 (100)	23 (92)	90 (87)	15 (83)	11 (100)
Q20	Have you experienced any cases in which organ transplantation was abandoned due to a COVID-19 infection even though organ recovery was possible?	Yes	12 (7)	1 (9)	1 (11)	0 (0)	9 (9)	1 (6)	0 (0)
		No	158 (89)	10 (91)	8 (89)	23 (92)	89 (86)	17 (94)	11 (100)
Q21	How do you handle patients who are candidates for organ transplantation during the COVID-19 pandemic? (Multiple answers allowed)	Be sure to perform chest CT	125 (70)	8 (73)	6 (67)	11 (44)	85 (83)	13 (72)	2 (18)
		Perform chest CT if the patient has some symptoms	16 (9)	1 (9)	0 (0)	5 (20)	3 (3)	3 (17)	4 (36)
		Be sure to screen for COVID-19 (PCR test, antigen test, etc.)	161 (90)	9 (82)	9 (100)	22 (88)	93 (90)	18 (100)	10 (91)
		Screen for COVID-19 (PCR test, antigen test, etc.), if there are chest CT findings or the patient has some symptoms	6 (3)	1 (9)	0 (0)	2 (8)	2 (2)	0 (0)	1 (9)
		Others	6 (3)	1 (9)	0 (0)	0 (0)	5 (5)	0 (0)	0 (0)
Q22	Have there been any changes in the post-transplant follow-up systems during the COVID-19 pandemic in your hospital?	No change	65 (37)	6 (55)	3 (33)	9 (36)	37 (36)	6 (33)	4 (36)
		The system has changed a little	80 (45)	5 (45)	6 (67)	11 (44)	45 (44)	9 (50)	4 (36)
		The system has changed considerably	29 (16)	0 (0)	0 (0)	5 (20)	18 (17)	3 (17)	3 (27)
		The system has all changed	3 (2)	0 (0)	0 (0)	0 (0)	3 (3)	0 (0)	0 (0)
Q23	If you answered "yes" to Q22, please explain how the post-transplant follow-up systems have changed. (Multiple answers allowed)	Outpatient visits for patients after transplantation were set to be performed at longer intervals	123 (69)	6 (100)	4 (80)	16 (76)	77 (94)	12 (92)	8 (89)
		Try to give more medicine than usual	66 (37)	4 (67)	3 (60)	9 (43)	44 (54)	4 (31)	2 (22)
		Examination are limited as much as possible	14 (8)	0 (0)	1 (20)	2 (10)	9 (11)	2 (15)	0 (0)
		Try to patients stay in the out-patients clinic as short as possible	51 (29)	2 (33)	3 (60)	5 (24)	35 (43)	4 (31)	2 (22)
		Try to make each other patients avoid contact	36 (20)	3 (50)	2 (40)	5 (24)	21 (26)	2 (15)	3 (33)
		Others	21 (12)	2 (33)	3 (60)	5 (24)	8 (10)	1 (8)	2 (22)

During the period of the questionnaire (December, 2020–January, 2021), transplant surgery was still suspended at seven centers (4%), all of which were kidney transplant programs. The reasons for resuming transplant surgeries (Q15) were as follows: reduction in the number of COVID-19 infections at the hospital (*n* = 9; 5%), lack of spread of COVID-19 infection confirmed in the hospital (*n* = 11; 6%), reduction in the number of COVID-19 infections in the local area (*n* = 29; 16%), establishment of an in-hospital medical care system and testing for COVID-19 (*n* = 51; 29%), restrictions on surgeries other than transplantation (*n* = 28; 16%), and surgeries re-established in accordance with the guidelines of the Japan Society for Transplantation (*n* = 34; 19%).

Of the centers with ongoing transplant surgery at the time of the questionnaire, 112 (63%) were providing transplant medical care without special restrictions, while 34 (19%) were limiting transplant surgery to cases considered difficult to postpone (Fig. 4d) (Q16). Depending on the future spread of the COVID-19 pandemic, only 30 centers (17%) answered that they would continue to provide transplant medical care without any restrictions, while 92 (52%) answered that they would consider suspending their transplant surgeries depending on the presence of COVID-19 in the ICU or hospital. Thirty-four centers answered that they would discontinue transplant surgeries in the event of nosocomial COVID-19 infection (Q17).

During the COVID-19 pandemic, 21 centers (12%) answered that they had been restricted from dispatching to organ recovery (Q18), and 14 centers (8%) answered that they had abandoned organ recovery efforts because of the COVID-19 pandemic (Q19). Even when organ recovery was possible, 12 centers (7%) reported that they had abandoned transplant surgery because of the COVID-19 pandemic (Q20).

Before any transplant surgery, 161 institutions (90%) performed a preoperative COVID-19 screening test of SOT recipients (Q21). A total of 112 centers (63%) answered that the post-transplant follow-up systems had changed in accordance with the COVID-19 pandemic (Q22), and most (123 centers, 69%) answered that follow-up outpatient visits after transplantation were set at longer intervals (Q23).

## Discussion

The findings of this study confirmed that the COVID-19 pandemic reduced the number of organ donations, especially from DCDs. In Japan, organ donation from DCDs was performed without the withdrawal of life-support including respiration, which sometimes forces the organ recovery team to wait a long time in the donor's hospital. This was considered why DCDs were so markedly reduced by the COVID-19 pandemic. Our questionnaire survey revealed the organ donation situation in Japan. In addition to a reduction in the number of organ donations, half of the transplant centers suspended transplant surgery, particularly abdominal organ transplant surgeries, because of the COVID-19 pandemic. While transplant surgeries were resuming in most institutions when the questionnaire was distributed, approximately 20% of the transplant centers limited SOT surgery to those patients whose prognosis would have been severely affected by postponing surgery, and about 10% of centers indicated that it was necessary to abandon organ recovery or SOT in some cases because of the COVID-19 pandemic. The present study confirmed that the COVID-19 pandemic has had a significantly negative impact on both organ donation and the performance of SOT.

In the United States, the number of patients registered as waiting for SOT and transplant surgery in April, 2020, was reported to have decreased in all United Network for Organ Sharing regions, and the mortality rate of these waiting patients had increased in more than half of the regions [34]. It was also reported that in March and April, 2020, when the COVID-19 pandemic began, the number of new wait-list patient enrollments, deceased-donor kidney transplants, and living-donor kidney transplants, fell below expectations by 18%, 24%, and 87%, respectively [35]. In Europe, the COVID-19 pandemic was reported to have had a similarly severe impact on transplant medical care, with the number of referrals of potential donors decreasing by 39% in the United Kingdom [36] and the number of potential deceased organ donors decreasing by 16% in comparison with previous years in France [37]. Conversely, in South Korea, (as in Japan), where the COVID-19 pandemic manifested relatively early with a less impact than in the United States and Europe, there was no significant change reported in the number of liver transplantations or kidney transplantations as of March and April, 2020 [38, 39], respectively, from the previous year, for both living donor transplants and BDD transplants. These findings showed that the ability to perform organ transplantation is dependent on the severity of the spread of COVID-19 in a given country; however, as these reports relate to the situation in spring 2020, subsequent reports on the overall situation of organ transplantation in 2020 are awaited.

Unfortunately, during the severe COVID-19 pandemic at the present time, in many countries, including Japan, available medical resources are likely to be assigned to countermeasures against the COVID-19 pandemic, necessitating a reduction in transplantation activity. In several countries, newly developed vaccines are improving the impact of the COVID-19 pandemic [40–45], and facilitating the rebuilding of a normal lifestyle. It is thought that the acquisition of herd immunity through vaccination will improve the survival of patients with organ failure who are awaiting transplantation and promote transplant medical care.

The present study had several limitations. The overall response rate to the questionnaire was 86%, which may be considered relatively high; however, the response rate of the kidney transplant centers was approximately 80%, which is slightly low. The questionnaire survey in this study was conducted from December, 2020 to January, 2021, when the background of the COVID-19 pandemic in Japan was in a state of flux. In particular, the number of COVID-19-positive patients in the third wave increased rapidly, and from January 2021, a state of emergency was declared in some cities, including Tokyo. The movement of people was greatly restricted and the situation was changing, which may have affected organ recovery; thus, the answers to our questionnaire survey might have varied greatly depending on the time of the response. Furthermore, the number of living donor organ transplantations was not mentioned in this paper, as the effects of the COVID-19 pandemic on the state of living transplantation in Japan is being investigated in another study.

## Conclusion

At the end of March, 2021, the number of patients infected with COVID-19 in the third wave of the pandemic began to decline. Although the COVID-19 pandemic in Japan is less severe than in other countries, it has had a large impact on the overall transplantation activity, suspending transplantation surgeries in 48% of the transplantation centers, including 78% of the kidney transplantation centers, and reducing the number of organ donations to 61% of the year-on-year average. This situation should be monitored closely.
